# Instructed Partnership Appreciation in Depression: Effects on Mood, Momentary Relationship Satisfaction, and Psychobiological Arousal

**DOI:** 10.3389/fpsyt.2020.00701

**Published:** 2020-07-30

**Authors:** Marco Warth, Martin Stoffel, Friederike Winter, Marc N. Jarczok, Corina Aguilar-Raab, Beate Ditzen

**Affiliations:** ^1^ Institute of Medical Psychology, Heidelberg University Hospital, Heidelberg, Germany; ^2^ Ruprecht-Karls University Heidelberg, Heidelberg, Germany; ^3^ Department for Psychosomatic Medicine and Psychotherapy, University Medical Center Ulm, Ulm, Germany

**Keywords:** depression, couple interaction, relationship, social interaction, stress response, cortisol, alpha-amylase

## Abstract

**Background:**

Depressive disorders are associated with attentional bias and social anhedonia. There is evidence supporting the hypothesis that depressed individuals participate less in potentially rewarding social situations and exhibit alterations in stress reactivity. With the present study, we aimed at investigating the affective and psychobiological response of couples with a depressed (female) partner in an instructed partnership appreciation task (PAT) that included positive and appreciative communication.

**Methods:**

In a quasi-experimental repeated-measures design, depressive couples (DCs)—i.e., the female partner being diagnosed with a depressive disorder—were compared to non-depressive couples (NDCs). Study outcomes were the PAT-induced changes in state mood, momentary relationship satisfaction, salivary cortisol, and salivary alpha-amylase. Additionally, we assessed psychometric baseline data on depression, relationship quality, social support, and chronic stress. Data was analyzed using multilevel modeling.

**Results:**

A total of 184 individuals from *N* = 47 DCs and *N* = 45 NDCs were included. DCs were characterized by higher depressiveness, lower relationship quality, less actually received social support from the partner, and higher chronic stress than NDCs. Manipulation checks led to the additional exclusion of two couples. Regarding mood, depressed women showed lower baseline scores and no significant differences in mood increase compared to non-depressed women (*p* = 0.107). Increases in relationship satisfaction were significantly stronger in the depressed group (*p* = 0.035). In addition, we found a significantly stronger cortisol increase in depressed women, but only if relationship duration was taken into account as a moderating factor (*p* = 0.022). No significant group differences were found for women’s amylase trajectories or for sex-dependent interaction effects on the couple level (all *p >* 0.05).

**Conclusions:**

Instructed engagement in positive couple interaction may require high effort and increased psychobiological arousal, but may finally result in emotional and social benefits in depressed women. While these findings encourage speculations about the therapeutic application of instructed partnership appreciation, more research is needed on the effectiveness of such interventions and on the moderating role of relationship duration in depression and couple functioning.

## Introduction

With an estimated incidence of 300 million cases worldwide, the World Health Organization’s Global Burden of Disease Study ranks depressive disorders as the single largest contributor to global disability (1, 2). In addition to common symptoms of anhedonia, poor concentration or sleep disturbances, depression can have a detrimental effect on social functioning and the quality of relationships. Moreover, depressive disorders were found to be accompanied by alterations in the neurobiological stress-regulatory systems, including the hypothalamic–pituitary–adrenal (HPA) axis and the autonomic nervous system (ANS). Hence, it seems crucial to take into account an integrated “bio-psycho-social” perspective—addressing psychobiological dysfunctions, subjective emotional and cognitive strain, and impaired social relationships equally—to approach a comprehensive understanding of depressive disorders (3).

### Social Dysfunction in Depression

Receiving promising support from neuroimaging studies, the social brain hypothesis has highlighted the importance of the social domain of human behavior and cognition. Depressive disorders were specifically proposed as an entity where the social constraints need to be taken more into account (4, 5). In general populations, a growing body of research has provided evidence for the health promoting effects of adaptive social relationships (6–8). In couple constellations, the physical health of one partner predicts the quality of life of the other, even after controlling for one’s own status (9, 10). Depressive patients, in contrast, were reported to benefit less from these health promoting effects (5). Moreover, there seems to be a bi-directional association between depression and relationship quality (11): On the one hand, relationship conflicts were proposed as a relevant contributor to depressiveness, on the other hand, symptoms of depression such as social withdrawal or loss of interest are a serious challenge for existing relationships (12, 13). Some authors proposed this association, in turn, to be moderated by relationship duration. Marital happiness was found to decline over the years (14, 15), while the risk for depressiveness is increased in long-term relationships (16, 17).

In particular, a substantial proportion of acute depressive episodes is accompanied by social anhedonia, i.e. the reduction of interest in or pleasure from social engagement (18). Previous research looked into both the internal processing and behavioral manifestations of social anhedonia. Regarding the first, a generally heightened focus on internal states was reported to reduce engagement with the social environment and to lead to interpersonal difficulties (18). In addition, a meta-analysis on eye-tracking data found that individuals suffering from depression spent significantly more processing time on dysphoric and less time on positive information than healthy controls (19). Moreover, studies suggest that this attentional maintenance bias transfers to socially relevant stimuli such as emotional facial expressions and is present in both acute and remitted forms of depression (19–21). These associations could be grounded in alterations of the reward system, with depressive individuals showing less motivation and capacity to respond to rewarding stimuli (19, 22).

With regard to behavior, a recent network analysis of RFID-based position tracking in a student camp revealed that that depressive symptoms were associated with a reduction of time spent in social interactions in general and particularly with friends, as well as with an increase of time spent with similarly depressed others (23). More specifically, depressed individuals showed impaired communication and interaction skills (24, 25) as well as difficulties in empathy and perspective taking (26). Social anhedonia manifest itself in reduced attempts to approach social situations (18). In general populations, women with high levels of depression were found to anticipate less positive response from social interaction and to engage less in approaching behavior (27). Reduced engagement in rewarding social interaction, in turn, impedes potential effects from positive social feedback (27). The described pattern of socially relevant depressive behavior may be due to self-serving biases including the tendency to avoid threatening social upward comparison (28), reduced attributed trustworthiness in interaction partners (25), and the fear of social rejection (5, 29).

For the majority of adults, a satisfying romantic relationship is the main source for social support (30) and a key determinant of quality of life (31). Unsurprisingly, the abovementioned social dysfunctions were also found in couple research. A recent longitudinal study found evidence for a possible causal effect of marital discord on the emergence of depressive symptoms (32). Moreover, more negative communication styles including accusation, hostility or aggression and less positive styles such as problem-solving behavior and self-revelation were observed in couples with depression than in control couples without depression (33, 34). These effects remained robust after controlling for general marital/relationship distress. In a study using a non-clinical sample, depressiveness in the female partner was associated with less empathic accuracy towards unpleasant feelings of the male partner (35). Moreover, individuals with higher depression scores underestimated the partner’s commitment and overestimated his/her negative behavior (36). Another series of studies suggests that the intimacy and trust of a relationship may buffer the use of these maladaptive emotion regulation strategies (37).

### Altered Stress Regulation in Couples With Depression

Both social isolation and depression in general are associated with decreased physical health. A lack of social connectedness was found to be a risk factor for immune dysfunction (38) and premature mortality (7, 8). On the other hand, meta-analyses revealed high marital quality as a predictor for general health (11). Physical touch and emotional intimacy from a romantic partner, in particular, were found to buffer cortisol response in healthy females in the Trier Social Stress Test (TSST) (39, 40) and in couples’ everyday life (41). Similarly, depression is associated with poor health outcomes in couples including the risk for cardiovascular diseases and general mortality (9). Stress and its underlying neuroendocrine, autonomic, and immune regulation processes have been introduced as a crucial mediator in the multi-directional association between depression, social functioning, and health (9). For instance, satisfying couple relationships buffer the adverse effects of stressful life events on the development and maintenance of physical and mental diseases, while marital conflict itself can serve as a powerful stressor and exacerbate depressive symptoms (38, 42).

Further, acute and recurrent depressive disorders seem to alter multiple biological stress-regulatory systems and the level of general arousal (43, 44). Besides inflammatory processes, research focused mainly on dysregulation of the HPA axis and ANS (9). Dysfunction in glucocorticoid regulation, particularly regarding the steroid hormone cortisol, is one of the most frequently studied phenomena in this context (45, 46). Altered circadian rhythms of cortisol release were associated with sleep disturbances, and increased cortisol secretion in the morning was found to be a risk factor for depressive diseases (47, 48). Moreover, meta-analytic syntheses showed generally elevated levels of cortisol secretion in depressed patients across multiple assessment methods (49), and an increase in reactivity towards psychosocial stressors, in particular (50). The magnitude and direction of effects, however, depends on moderating variables such as sex, diagnosis, type of stressor, and measurement plan. Cortisol release in response to the TSST, for instance, was blunted in women with remitted major depression compared to healthy controls, but not in men (51). A longitudinal study showed cortisol levels to be associated with the persistence of depressive symptoms (52). Moreover, depressed women showed weaker associations between morning cortisol increases and the occurrence of social interactions and perceived these interactions as more negative than healthy women (53). Regarding romantic couples, women’s depression scores were positively related to their partners cortisol output (54) and high depressiveness in women predicted an attenuated cortisol response after a relationship conflict discussion with the partner in another recent study. In male participants, however, cortisol levels were generally elevated if depression scores were high (55). Hence, the question of HPA hypo- vs. hyperactivity in couples with depression is still subject to controversy, and it seems crucial to take sex differences into account.

Recent research has emphasized the complex and dynamic interplay between the HPA axis and the ANS in the regulation of chronic and acute stress, and it has been recommended to monitor both systems simultaneously in the study of the human stress response (56). Besides feasible cardiovascular, autonomic markers such as heart rate variability, salivary alpha-amylase (sAA) has been introduced as a promising biomarker of sympathetic arousal (57–59). sAA is an enzyme produced by the parotid glands in response to acute adrenergic innervation. It has thus been studied as a proxy for the sympatho-adreno-medullary (SAM) branch of the ANS in stress research (57, 60–62). Previous studies showed an sAA increase in response to the TSST (63), after pharmacological stimulation of adrenergic receptor systems (61) and after different psychologically or physically induced arousal paradigms (59). A systematic review identified substantial alterations in sAA-reactivity in the context of mental illness including depression (64). Moreover, an elevated release of sAA was associated with increased feelings of depression and shame in general populations (57, 65). Individuals with a current episode of depression showed higher levels of sAA than remitted patients (66) and an elevated sAA reactivity to an electrical stimulation stressor compared to healthy controls (67).

### Rationale and Aim of the Study

Taking into account the abovementioned complex dynamics, we followed an integrated approach to the understanding of social behavior in depressed couples. This study compared the psychological and psychobiological response of depressed and non-depressed romantic couples in an instructed partnership appreciation task (PAT) that included positive and appreciative communication. The rationale for the use of the PAT in our study was influenced by two directions of previous literature on instructed social interactions between romantic partners, namely couple therapy (68) and experimental mood induction tasks (69). Inspired by couple therapy research, we developed a list of positively connoted conversation topics and asked couples to express appreciation for each other and to share positive experiences with the idea to increase positive reciprocity (70, 71). At the same time, this task was intended to induce positive mood in a naturalistic couple setting [as opposed to e.g. mood induction by auditory or visual stimuli, (69)]. The hypothesized differences in the psychobiological response are based on the abovementioned literature on the connection between depression and the responsiveness of stress-reactive systems in social situations (53). I.e. both cortisol and sAA were described in previous literature as markers of physiological arousal in response to stressful situations (44, 58), and both may show altered functioning over the course of a depressive disorder (53, 72). We expected that—due to social anhedonia and the evident phenomena of positive interactions occurring less frequently in everyday life and being perceived as less pleasant (23, 53)—engaging in an instructed PAT would require high internal resources and induce (or alter) physiological arousal in depressed individuals who would usually tend to avoid PAT-like situations.

Hence, with the observation of (close to) naturalistic behavior between real-life partners and the emphasis on positive instead of negative interaction, we aimed at extending previous research that rather focused on conflict behavior, non-intimate laboratory stressors, or non-interpersonal mood induction. The integrated monitoring of psychobiological arousal was a novel aspect in this study, and the general hypothesis was that couples with depression, and the depressed female index-patients in particular, would benefit less from instructed positive couple interaction, in comparison to healthy controls. We expected this pattern to lead to different changes in the ratings of state mood and momentary relationship satisfaction and to different HPA and SAM activation trajectories in response to the PAT. The study hypotheses are specified below (section *Multilevel Modeling for Hypotheses Testing*).

## Materials and Methods

### Study Design and Ethics

In a quasi-experimental, repeated-measures design, we compared so-called “depressive couples” (DCs; i.e. couples with the female partner being diagnosed with a depressive disorder) to non-depressive couples (NDCs) with regard to their psychobiological stress response in the PAT. This study received approval by the Ethics Committee of the Medical Faculty at Heidelberg University (S-021/2016). All participants gave written informed consent in accordance with the declaration of Helsinki.

The present analysis is based on the first part of the SIDE (Social Interaction in Depression) study series. The SIDE studies contained a cross-sectional, first part in which self-report, psychobiological, and eye-tracking data was collected from DCs and NDCs, and an interventional, second part where participating DCs were randomized to either a 10-week Cognitively Based Compassion Training (CBCT^®^) for couples or to a control treatment. Procedures and methods of this randomized controlled trial (RCT) can be found in the published study protocol (73). No protocol was pre-registered for the cross-sectional part, which is reported here, but many of the present methods (e.g. sample size calculation, outcome measures) were influenced by the consideration to later conduct the RCT with partly overlapping samples (NDCs were not included in any subsequent study). The reasons for the overlap in methods in the SIDE studies were to address well-known recruitment challenges in clinical trials in couples with psychopathology, and the assumption that financial incentives alone would not ethically justify the required assessment effort in some severely distressed couples.

### Participants

Recruitment strategies for couples in both groups involved newspaper advertising, posters and flyers in public places, advertising in public transport, social media, and university mailing lists. For the recruitment of DCs, we additionally contacted registered doctors, psychiatric and psychosomatic clinics, as well as regional outpatient centers for counseling and psychotherapy. Due to the abovementioned sex differences with regard to stress-reactivity in depression, the study focused on the inclusion of female patients suffering from depression and their romantic partners. Inclusion and exclusion criteria for DCs and NDCs are listed in **Table 1**.

**Table 1:** Inclusion and exclusion criteria.

	Inclusion	Exclusion
**DCs—Women**	SCID diagnosis: Depressive episode or recurrent depressive disorder (F32.X, F33.X, F34.1) HDRS score ≥ 12 Age ≥20 years In a romantic, heterosexual relationship for ≥2 years	Psychotic symptoms Bipolar disorder Acute suicidal tendency Present substance abuse
**DCs—Men**	Age ≥20 years In a romantic, heterosexual relationship for ≥2 years	Psychotic symptoms Bipolar disorder Acute suicidal tendency Present substance abuse
**NDCs—Women**	Age ≥20 years In a romantic, heterosexual relationship for ≥2 years	Any current psychiatric diagnosis (SCID) HDRS score ≥12
**NDCs—Men**	Age ≥20 years In a romantic, heterosexual relationship for ≥2 years	Any current psychiatric diagnosis (SCID) HDRS score ≥12

DCs, depressive couples; NDCs, non-depressive couples; SCID, Structured Clinical Interview of the Diagnostic and Statistical Manual of Mental Disorders; HDRS, Hamilton Depression Rating Scale.

### Procedures and Tasks

This study was conducted at the Institute of Medical Psychology at Heidelberg University Hospital in Germany. Interested couples initially participated in a brief, standardized telephone interview for a first screening of eligibility (e.g. relationship status and duration). Afterwards, couples were invited to our Social Interaction Lab for a laboratory assessment on two consecutive days. On lab day 1, participants were informed about the study goals, procedures, potential risks and benefits, and were asked to sign the consent form. Participants were then screened for the presence of any mental disorder and depression in particular by use of the Structured Clinical Interview for DSM-IV (SCID) and the Hamilton Depression Rating Scale (HDRS) (74, 75). While one partner was interviewed, the other was asked to fill out questionnaires on demographic and health data (including information on education, income, employment, physical activity, health status, and on menstrual cycle for female participants) and a number of clinical psychometric scales (see *Additional Clinical Measures*). Questionnaire data was collected with a tablet computer and the online software SoSci Survey (76).

On lab day 2, we carried out an interview and measurements on possible confounding variables recommended for cortisol research including body mass index (BMI), current medication, caffeine/alcohol/nicotine intake, and physical exercise (77). Afterwards, participants received an instruction for the PAT. Couples were seated on opposite sides of a table and read a list with 23 conversation themes (e.g. attractiveness, trust, tolerance). Themes were adopted from the problem list used in research on couple conflict (71), but were modified to have a positive instead of negative connotation (e.g. loyalty instead of jealousy). Couples were instructed to speak only about positive content, to be supportive and appreciative, and to switch to another theme if they noticed any upcoming conflict or unpleasant feelings. The experimenter then left the room for 10 min, while the partners were asked to start the interaction. Conversations were video-taped and rated for adherence to the instructions by three independent, blinded research assistants on a scale ranging from (1) *very negative* to (5) *very positive*. Instruction materials for the PAT can be found in the **Supplemental Materials** of this publication.

**Figure 1:**
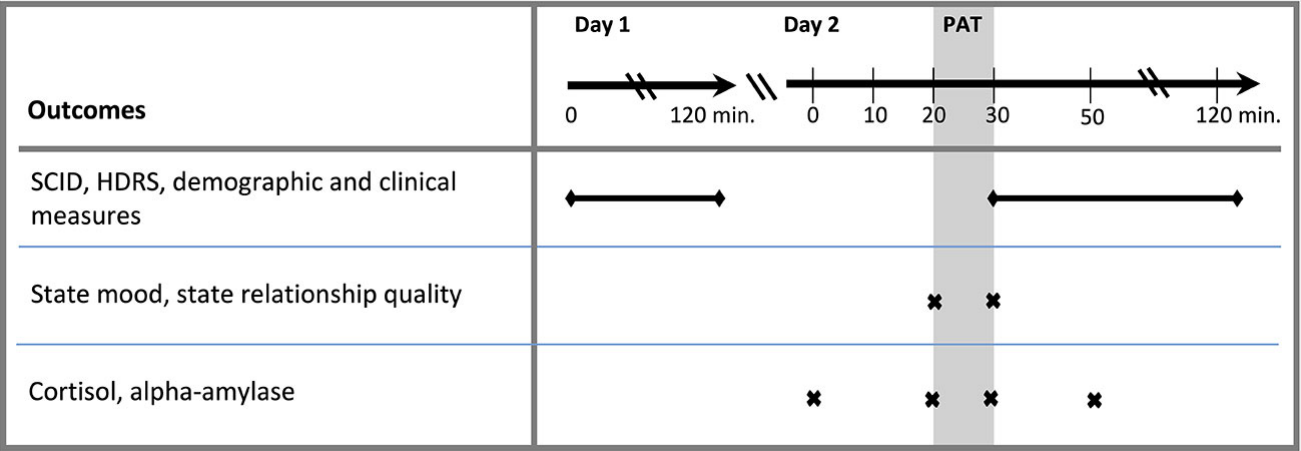
Assessment plan. SCID, Structured Clinical Interview of the Diagnostic and Statistical Manual of Mental Disorders; HDRS, Hamilton Depression Rating Scale; PAT, positive social interaction.

We collected a total of four saliva samples from each participant: (T1) 20 min before PAT, (T2) immediately before PAT, (T3) immediately after PAT, (T4) 20 min after PAT (**Figure 1**). Psychobiological assessments on lab day 2 were carried out at standard times in the afternoon between 2 p.m. and 5 p.m. Additionally, participants were asked to fill out a brief questionnaire on acute mood states and a single-item scale on perceived relationship satisfaction at that moment, immediately before (T2) and after the PAT (T3). The post-PAT (T3) assessment also contained a single item asking for the individual’s perception of the previous conversation on a 5-point scale ranging from *(1)* *very negative* to *(5) very positive*, for the purpose of manipulation check. After the PAT, participants completed the second part of the tablet-based psychometric assessment.

### Outcomes

The study outcomes encompassed PAT-related changes in state mood (MOOD) and momentary relationship satisfaction (RELSAT), both measured pre- (T2) to post PAT (T3). Moreover, we repeatedly measured the HPA and SAM response to the PAT *via* salivary cortisol (sCORT in ng/ml; T1–T4) and salivary alpha-amylase (sAA output in U/min; see (78); T1–T4).

#### State Mood and Momentary Relationship Satisfaction Scale

Participants rated their state mood on three bipolar scales (1–5) based on the Multidimensional Mood Questionnaire’s (MDBF) mood subscale (79, 80): *annoyed–in a good mood, content–discontent, happy–unhappy*. Item responses were averaged for calculation of a total score (MOOD), with higher values indexing more positive mood. Additionally, participants were asked for their momentary perception of state relationship satisfaction (RELSAT) from (1) *very dissatisfied* to (5) *very satisfied*. This single-item assessment was adapted from the Relationship Assessment Scale (RAS), which showed adequate internal consistency and validity in previous studies (81, 82). Both scales were assessed once before and once after the PAT. Modification of existing scales was necessary to enable brief assessments and change sensitivity in the very short measurement time course and has been shown to be feasible in a previous study (79).

#### Cortisol and Alpha-Amylase Assessment

We used the passive drool method and SaliCab^®^ tubes (RE69985, IBL, Hamburg, Germany) to collect four whole saliva samples per participant. Participants were asked to collect saliva for one minute and to salivate through a plastic straw into the collecting tube. Saliva samples were stored at -80°C until laboratory analysis. sCORT was analyzed using a commercially available enzyme-linked immunosorbent assay (DES6611; Demeditec Diagnostics, Kiel, Germany) according to the manufacturer’s protocol. sAA was analyzed using a kinetic colorimetric kit with reagents from Roche (Roche Diagnostics, Mannheim, Germany). Biological data were analyzed in the stress biomarkers lab at the Institute of Medical Psychology, Heidelberg. The intra-assay coefficient of variation (CV) was 3.35% for sCORT and 3.36% for sAA. The inter-assay CV was 7.20% for sAA and 6.28% for sCORT.

#### Additional Clinical Measures

For the purpose of sample characteristics description and statistical control of unintended variability in the outcome data, several psychometric scales were assessed once at either lab day 1 or 2. A complete list of all scales collected in the SIDE studies can be found in the RCT’s protocol (73). The following scales were used in the present study: The Patient Health Questionnaire (PHQ-9), the Partnership Questionnaire (PFB), the Berlin Social Support Scale (BSSS), and the Trier Inventory for Chronic Stress (TICS).

The PHQ-9 is a brief, self-report screening tool for depression severity consisting of nine items on a 4-point scale (83). Validation studies reported high reliability and an acceptable one-factor model fit for the German version (84). The PFB is a diagnostic instrument for the assessment of partnership quality frequently used in German-speaking countries, with adequate internal consistency and validity (85, 86). The questionnaire uses 30 items on a 4-point scale to measure partnership related behavior and attitudes on the subscales “quarreling”, “tenderness”, and “similarity/communication”. The BSSS measures social support in the course of a stressful event (e.g. coping with a disease). Responses to 34 items on a 4-point scale can be aggregated to one of six available subscales (perceived, actually received and actually provided support, need for support, support seeking, protective buffering). Reliability and validity were reported to be sufficient for the BSSS (87). Moreover, to measure the presence of chronic stress in our participants, we used the 12-item (0–4) screening subscale of chronic stress (SSCS) of the TICS. Adequate psychometric properties were reported in a German validation study (88).

We used total sum scores of all scales for sample description purposes, except for the BSSS, which does not allow for calculation of a total score. Here, we used the “actually received social support” subscale (calculated as mean), as it asks specifically for support by a romantic partner (87). For all reported scales, higher numeric values indicate a higher score on the labeling construct: high depression (PHQ-9), partnership quality (PFB), social support (BSSS), and chronic stress (TICS).

### Analytical Plan

#### Preliminary Analysis and Handling of Covariates

With regard to the manipulation check, observer-ratings of the PATs were averaged across raters. For both the self- and observer-ratings, we calculated means and 95% confidence intervals (CIs), first for the entire sample and then for study groups separately (DCs and NDCs). Couples, whose self-ratings and averaged observer-ratings were all below 3, were considered “non-compliant” to the PAT instructions, and thus, were deleted from the outcome models.

Before calculating the outcome models, a number of potential confounders and moderators were tested for their association strength with the study outcomes. These variables were derived from guidelines on stress biomarker research (77), from the clinical scales used in this study (PHQ-9, PFB, BSSS, TICS), and from preselected demographic/health screening variables that were relevant to the research question (e.g. blood pressure, age, relationship duration, medication intake). Balancing between statistical control and model convergence, we decided to consider caffeine intake (no/yes), smoking (no/yes), and BMI (in kg/m^2^) as time-invariant covariates for the psychobiological outcomes, and age for all outcomes. Since associations between relationship duration (RELDUR) and the study outcomes were particularly consistent, we chose to explore its potentially moderating role in the course of multilevel modeling. Additionally, associations of RELDUR with other relevant study data were exploratively analyzed by Pearson product-moment correlations and 95% CIs calculated *via* Fisher’s *z* (back-)transformation. Further statistical procedures and handling of predictor variables are described in the following paragraph.

#### Multilevel Modeling for Hypotheses Testing

Given the nested structure of the data (measurements nested in individuals and individuals nested in dyads), statistical analysis was conducted using multilevel modeling (89, 90). To test the study hypotheses, we decided to follow a two-step analytical strategy: In the first step, the primary hypotheses (see below) were tested in a women-only data subset, eliminating the couple level. This analysis was of primary interest as we hypothesized differences in the PAT response between female index patients and non-depressed female controls. In a secondary step, we included the data of male partners, but eliminated the measurement level (TIME) by collapsing repeated measures into a change score or area under the curve with respect to increase (AUCi). Change scores were calculated by subtracting pre from post scores for state mood (MOOD_d) and relationship satisfaction (RELSAT_d). AUCi’s were computed for sCORT and sAA according to standard procedures in psychoneuroendocrine research (91). In addition to outcome hypotheses testing, AUCi’s were used for illustrative purposes in the graphical outputs. If single measurements were missing within one person, they were imputed by use of the R package Amelia II (92) before calculation of the AUCi’s.

Hence, multilevel models were built to test the following focal predictors and hypothesis:

1. Primary hypotheses: Women’s PAT response (with regard to MOOD, RELSAT, sCORT, and sAA) is moderated by GROUP * TIME (Models 1 to 4)
2. Exploratory hypotheses: GROUP * TIME effects in women are moderated by relationship duration (GROUP * TIME * RELDUR)
3. Secondary hypotheses: PAT response of all participants (with regard to MOOD_d, RELSAT_d, sCORT AUCi, and sAA AUCi) is moderated by SEX*GROUP (Models 5 to 8)
4. Exploratory hypotheses: SEX * GROUP effects in all participants are moderated by relationship duration (SEX * GROUP * RELDUR)

Models were fitted in the statistical environment R (93) *via* the “lme” function of the “nlme” package (94) with a restricted maximum likelihood method of estimation (REML). The distribution of every outcome variable was examined. In case non-normality became evident, transformation techniques were applied, given that this helps to approximate normality of the model residuals. All continuous predictors, except TIME (0 to 1 for MOOD and RELSAT, 0 to 3 for sCORT and sAA) were centered on their grand mean. Dichotomous predictors were entered as factors. To account for the nested structure of the data and to minimize standard errors (95), random intercepts were added in each model. Random slopes were only considered for models with more than two lower-level units nested in higher level units (Models 3 and 4). We graphically assessed each final model for violations of central model assumptions regarding the distribution of residuals and random effects (96).

To test hypotheses 1.a, we built two-level models with TIME nested in individuals (women only). Both, sCORT and sAA data were positively skewed. To enable an approximate normality of the model residuals, both were transformed to the natural logarithm. Thereafter, outliers beyond three standard deviations of the mean were excluded. In the process of model fitting, we allowed the effect of time to vary across individuals only in the sCORT model, since this provided the best model fit as indicated by likelihood ratio tests for nested models as well as by the Bayesian information criterion (BIC). For testing of hypothesis 2.a, we built two-level models with individuals (all participants) nested in couples for the composite outcomes MOOD_d, RELSAT_d, sCORT AUCi, and sAA AUCi. Only MOOD_d was found to be positively skewed and was transformed to the natural logarithm (adding 5 as a constant first, because negative change scores would have been transformed to NA otherwise). The potentially moderating role of relationship duration was explored in all models (Models 1–8, hypotheses 1.b and 2.b). Only if the focal predictor in these models was statistically significant, final models including this interaction effect are reported.

#### Sample Size

Sample size calculations for the SIDE studies were tailored for the conduction of the subsequent RCT that would further include the DCs who participated in the present study. Analyses with G*Power (97) were described in the study protocol and revealed an optimal total sample size of *N* = 50 DCs, accounting for assumed attrition (73). In the present study, we aimed at recruiting an equal amount of *N* = 50 additional NDCs for the comparison of PAT responses. Power analyses showed that this sample size would allow us to detect small-sized effects (> *f* = 0.1) between DCs and NDCs in a repeated-measures design with *k* = 4 observations, a correlation between repeated-measures of ρ = 0.6, α = 0.05, and (1 − β) = 0.8 (97). Sample size calculation for multilevel modeling is more complex, but it is reasonable to assume that the G*Power analyses represent a conservative estimate, as previous simulation work has shown that a sample of *n* ≥50 subjects on level-2 allows for unbiased estimates of model coefficients, standard errors, and variance components (98).

## Results

### Sample Characteristics and Manipulation Check

A total of *N* = 116 heterosexual couples and *n* = 232 individuals were recruited (*N* = 65 DCs and *N* = 51 NDCs). *N* = 24 couples were excluded as they did not meet the requirements with regard to the presence or non-presence of a depressive diagnosis as defined in **Table 1**, or because no biodata was available at all. This resulted in a total of *n* = 184 individuals from *N* = 47 DCs and *N* = 45 NDCs to be included in the study. Additional individual data points were excluded in the course of psychobiological data preparation (see analysis sections and tables). With an overall mean of *M* = 4.26 (*CI* = [4.13; 4.39]) the total sample rated the PAT as positive on average. This was true for both DCs (*M* = 4.11, *CI* = [3.93; 4.29]) and NDCs (*M* = 4.42, *CI* = [4.24; 4.60]). The observer-based manipulation checks revealed similar results: Blinded raters on average perceived the PAT as positive (*M* = 4.22, *CI* = [4.03; 4.42]), and the difference between study groups was small in magnitude (DCs: *M* = 4.15, *CI* = [3.87; 4.43]; NDCs: *M* = 4.31, *CI =* [4.04; 4.58]). Interaction behavior in two couples (1 DC and 1 NDC), however, received ratings lower than 3 in both the self- and observer-ratings, leading to subsequent exclusion of this data from the outcome models.

**Figure 2:**
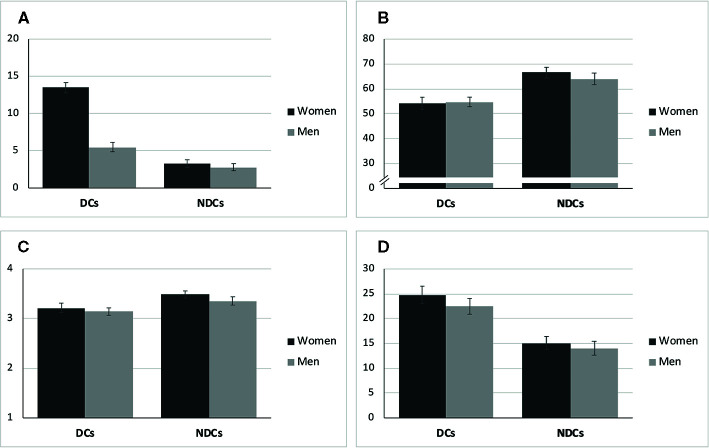
Means and standard errors of psychometric scales at baseline. **(A)** Depression (PHQ-9, Range: 0-27). **(B)** Relationship Quality (PFB, Range: 0-90). **(C)** Social Support (BSSS, Range: 1-4). **(D)** Chronic Stress (TICS, Range: 0-48). DCs, depressive couples; NDCs, non-depressive couples; PHQ-9, Patient Health Questionnaire; PFB, Partnership Questionnaire; BSSS, Berlin Social Support Scales (actually received support); TICS, Trier Inventory for Chronic Stress (screening subscale).

As **Table 2** shows, the study groups differed with regard to both age and relationship duration. DCs on average were *M* = 42.5 (*SD* = 14.8) years old and in the relationship for *M* = 11.3 (*SD* = 10.5) years, while NDCs were *M* = 36.7 (*SD* = 17.3) years old and in the relationship for *M* = 9.0 (*SD* = 11.9) years. Hence, both variables were considered potential covariates in the subsequent analyses. None of the included men in the DCs was diagnosed with a current form of depression *via* SCID. *N* = 7, however, had a HDRS rating ≥12. Moreover, *N* = 7 men in the DCs, *N* = 4 men in the NDCs, and *N* = 6 women in the NDCs reported a lifetime history of depression (fully remitted). **Figure 2** illustrates sex and group differences with regard to clinically relevant measures. As expected, women in the DCs had the highest PHQ-9 scores, but their female partners also reported moderately elevated depressiveness with an average of *M* = 5.51 (*SD* = 4.33) compared to the NDCs. Moreover, both partners in the DCs reported lower overall relationship quality (PFB) and actually received social support by the partner (BSSS) than NDCs (**Figures 2B, C**). A similar pattern of baseline differences occurred for the assessment of chronic stress with the TICS (**Figure 2D**): Both male and female partners indicated a higher stress level, if they belonged to the DCs group compared to NDCs, while sex-dependent differences within study groups on clinical measures other than the PHQ-9 were rather small.

**Table 2:** Descriptive statistics of sample characteristics and outcome data.

	Sex	DCs		NDCs	
		*N*	*M (SD)*	*N*	*M (SD)*
**Age**	**Women**	46	41.24 (14.13)	44	34.95 (16.38)
	**Men**	46	43.98 (15.84)	44	37.09 (17.60)
**Relationship duration in years**	**Women**	46	11.27 (10.80)	44	8.84 (12.21)
	**Men**	46	11.17 (10.64)	44	8.80 (11.94)
**Depression (PHQ-9)**	**Women**	46	13.54 (4.72)	44	3.00 (3.12)
	**Men**	46	5.33 (4.19)	44	2.84 (3.23)
**Relationship quality (PFB)**	**Women**	45	54.20 (16.75)	44	67.41 (13.45)
	**Men**	45	54.89 (13.16)	43	64.95 (14.37)
**Social support (BSSS)**	**Women**	44	3.23 (0.63)	43	3.48 (0.49)
	**Men**	44	3.15 (0.51)	44	3.37 (0.55)
**Chronic stress (TICS)**	**Women**	44	24.50 (11.74)	43	14.86 (8.62)
	**Men**	44	22.16 (10.56)	44	13.57 (8.89)
**State mood (MOOD)—Pre PAT**	**Women**	46	3.28 (0.83)	44	4.19 (0.71)
	**Men**	46	3.88 (0.63)	44	4.19 (0.79)
**State mood (MOOD)—Post PAT**	**Women**	46	3.91 (0.80)	44	4.55 (0.62)
	**Men**	46	4.20 (0.69)	44	4.53 (0.68)
**Momentary relationship satisfaction (RELSAT)—Pre PAT**	**Women**	46	3.57 (1.17)	44	4.45 (1.00)
	**Men**	46	3.83 (0.97)	44	4.55 (0.90)
**Momentary relationship satisfaction (RELSAT)—Post PAT**	**Women**	46	4.09 (1.07)	44	4.75 (0.53)
	**Men**	46	4.30 (0.70)	44	4.45 (1.13)
**sCORT_1**	**Women**	44	3.33 (1.52)	44	3.12 (1.96)
	**Men**	44	3.61 (1.72)	44	3.76 (2.53)
**sCORT _2**	**Women**	45	3.30 (1.45)	44	3.08 (1.50)
	**Men**	45	3.88 (1.89)	43	3.89 (2.32)
**sCORT _3**	**Women**	45	3.80 (2.81)	43	3.26 (1.91)
	**Men**	45	4.01 (2.18)	44	4.28 (2.96)
**sCORT _4**	**Women**	45	3.07 (1.86)	44	3.03 (2.00)
	**Men**	43	3.63 (2.13)	43	3.67 (2.37)
**sAA_1**	**Women**	43	69.92 (104.83)	44	74.40 (104.25)
	**Men**	44	98.28 (111.88)	44	80.87 (76.85)
**sAA_2**	**Women**	43	102.10 (184.69)	41	58.36 (39.92)
	**Men**	43	116.39 (162.32)	42	95.39 (89.36)
**sAA_3**	**Women**	43	114.17 (101.24)	43	112.57 (171.28)
	**Men**	44	137.50 (174.29)	44	104.11 (107.15)
**sAA_4**	**Women**	43	91.99 (91.83)	42	91.76 (93.97)
	**Men**	44	113.69 (130.18)	42	100.97 (99.14)
**sCORT AUCi**	**Women**	45	4.72 (59.73)	44	0.14 (56.68)
	**Men**	45	7.12 (61.85)	41	10.43 (64.55)
**sAA AUCi**	**Women**	44	885.11 (2235.90)	40	739.00 (2269.15)
	**Men**	43	797.96 (2901.10)	41	860.42 (2484.32)

M, mean; SD, standard deviation; PHQ-9, Patient Health Questionnaire; PFB, Partnership Questionnaire; BSSS, Berlin Social Support Scales—actually received support; TICS, Trier Inventory for Chronic Stress—screening subscale; PAT, Partnership Appreciation Task; DCs, depressive couples; NDCs, non-depressive couples; sCORT, salivary cortisol (in ng/ml); sAA, salivary alpha-amylase (in U/min); AUCi, area under the curve with respect to increase.

### PAT Response in Depressed vs. Non-Depressed Women

**Figure 3:**
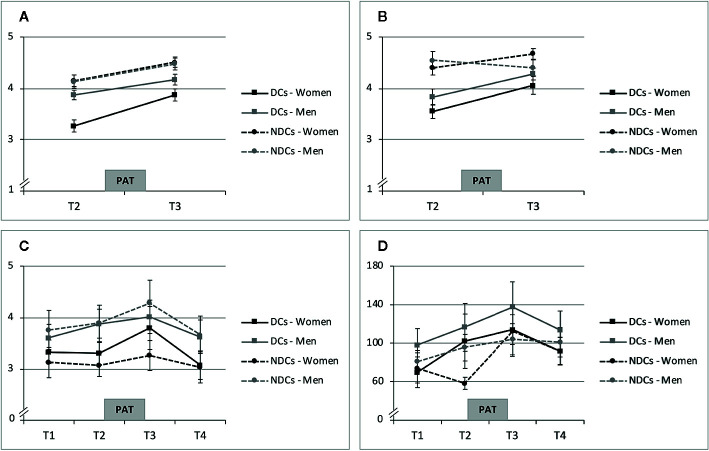
Means and standard errors of PAT response. **(A)** State Mood (Range 1-5). **(B)** Momentary Relationship Satisfaction (Range: 1-5). **(C)** Cortisol (sCort in ng/ml). **(D)** Alpha-Amylase (sAA in U/min). DCs, depressive couples; NDCs, non-depressive couples, PAT, partnership appreciation task.

**Table 2** includes means and standard deviations of all study outcomes (sCORT, sAA, MOOD, RELSAT), and trajectories of raw data means and standard errors over the course of the PAT are shown in **Figure 3**. Women in both groups showed increases in MOOD and RELSAT after the PAT. Baseline means were lower and mean increases were stronger in depressed women for both variables (**Figures 3A, B**). The tested TIME * GROUP effect was statistically significant for RELSAT (*p* = 0.035), but not for mood (*p* = 0.107). Hence, depressed women’s momentary relationship satisfaction increased significantly stronger, while the between-group differences in MOOD slopes over time were in the same direction but failed to reach significance. Relationship duration was not a significant moderator of MOOD or RELSAT change in women (both *p >*0.050) and was therefore not included in the final Models 1 and 2 (**Table 3**).

**Table 3:** Multilevel modeling of outcome data (observations nested in individuals), women only.

Fixed effects	Model 1: MOOD		Model 2: RELSAT		Model 3: sCORT		Model 4: sAA	
	Est.	*p*	Est.	*p*	Est.	*P*	Est.	*p*
INTERCEPT	**4.135**	**<0.001**	**4.484**	**<0.001**	**0.988**	**<0.001**	**3.821**	**<0.001**
TIME (0, 1, 2, 3)	**0.364**	**<0.001**	**0.210**	**0.038**	−0.012	0.525	**0.130**	**0.004**
GROUP (0 = NDCs, 1 = DCs)	−**0.725**	**<0.001**	−**0.708**	**<0.001**	0.030	0.762	−0.173	0.454
RELDUR (years)	–		–	–	0.000	0.957		
AGE (years)	−**0.017**	**<0.001**	−**0.017**	**0.001**	0.006	0.240	0.013	0.078
CAFFEIN INTAKE (0 = no, 1 = yes)	–		–	–	0.105	0.330	0.004	0.986
SMOKING (0 = no, 1 = yes)	–		–	–	0.260	0.143	0.163	0.659
BMI (kg/m^2^)	–		–	–	0.011	0.378	−0.010	0.697
TIME * GROUP	0.186	0.107^a^	**0.302**	**0.035^a^**	−0.033	0.214^a^	0.014	0.823^a^
TIME * RELDUR	–	–	–	–	−0.001	0.689	–	–
GROUP * RELDUR	–	–	–	–	−0.008	0.320	–	–
TIME * RELDUR * GROUP	–	–	–	–	**0.005**	**0.022** ^b^	–	–
**Random effects (variances)**								
INTERCEPT	0.322	–	0.428	–	0.164	–	0.687	–
TIME	–	–	–	–	0.010	–	–	–
Residual variance	0.143	–	0.213	–	0.023	–	0.405	–
BIC	361.599	–	413.504	–	232.554	–	892.014	–
Number of observations	177	–	173	–	340	–	332	–
Number of individuals	89	–	87	–	86	–	85	–

MOOD, state mood; RELSAT, momentary relationship satisfaction; sCORT, salivary cortisol (in ng/ml); sAA, salivary alpha-amylase (in U/min); RELDUR, relationship duration; BMI, body mass index; Est., Estimate; BIC, Bayesian information criterion; bold effects were statistically significant on the level of p <0.05; ^a^tested in hypothesis 1.a; ^b^tested in hypothesis 1.b.

**Figure 4:**
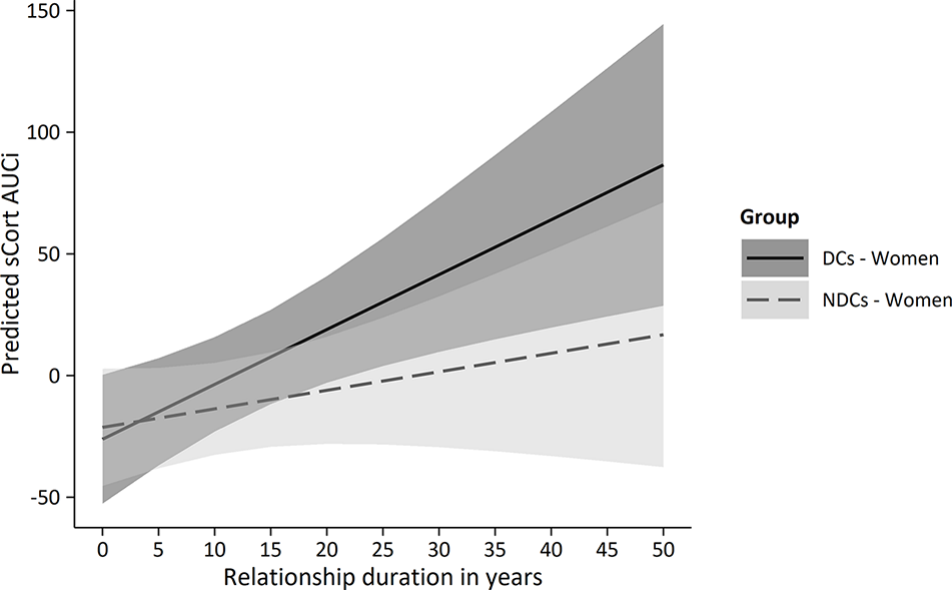
Predicted sCORT_AUCi by group and relationship duration in women. PAT, partnership appreciation task DCs, depressive couples; NDCs, non-depressive couples; sCORT, salivary cortisol (in ng/ml); AUCi, area under the curve with respect to increase.

Averaged sCORT trajectories of women in the NDCs group showed little change over time, while depressed women’s sCORT levels, in contrast, particularly increased from pre-PAT (T2) to post-PAT (T3; **Figure 3C**). Multilevel modeling showed that sCORT increases were significantly stronger in depressed women, but only if relationship duration was taken into account (**Table 3**). Hence, while we did not find a significant TIME * GROUP effect (*p* = 0.214), the three-way interaction TIME * GROUP * RELDUR was statistically significant (*p* = 0.022), indicating that the higher sCORT increase in depressed females was particularly pronounced in longer-term relationships. This effect is illustrated in **Figure 4**, where the sCORT AUCi was used as the outcome for illustrative purposes.

sAA increased from T1 to T3 and decreased after the PAT in depressed women (**Figure 3D**). Non-depressed women’s trajectories revealed comparable mean values at T1, T3, and T4, but a lower score at T2. Multilevel modeling showed a significant sAA increase in response to the PAT in all women regardless the study group (TIME, *p* = 0.009). We did not find a significant TIME * GROUP interaction (*p* > 0.050), however, and RELDUR was not a significant moderator in this analysis (*p >* 0.050) and was therefore not included in the final Model 4 (**Table 3**).

### Sex Differences in Depressed vs. Non-Depressed Couples’ PAT Response

State mood increases were observed in all study groups including men. In DCs, men’s MOOD levels were higher than those of their female partners (**Figure 3A**). Concerning RELSAT, both men and women in the DCs reported lower scores than NDCs, and between-group differences decreased after the PAT. Men in NDCs had the highest initial ratings and they were the only subgroup showing a slight decrease in RELSAT (**Figure 3B**). Models 5 and 6 in **Table 4** present the estimates and significance values with regard to the moderating role of sex in MOOD_d and RELSAT_d group differences. The tested SEX * GROUP effects failed to reach significance in both the change scores of state mood and momentary relationship satisfaction (MOOD_d, RELSAT_d, both *p >* 0.050).

**Table 4:** Multilevel modeling of outcome data (individuals nested in couples), all participants.

Fixed effects	Model 5: MOOD_d		Model 6: RELSAT_d		Model 7: sCORT AUCi		Model 8: sAA AUCi	
	Est.	*p*	Est.	*p*	Est.	*p*	Est.	*p*
INTERCEPT	**1.670**	**<0.001**	0.116	0.253	−1.793	0.877	650.086	0.100
SEX (0 = men, 1 = women)	0.004	0.817	0.113	0.419	−12.845	0.264	−55.720	0.885
GROUP (0 = NDCs, 1 = DCs)	−0.005	0.818	0.247	0.083	3.839	0.748	363.831	0.391
AGE (years)	0.000	0.851	0.005	0.111	**−0.569**	**0.049**	−8.256	0.451
CAFFEIN INTAKE (0 = no, 1 = yes)	–	–	–	–	15.455	0.128	−328.631	0.344
SMOKING (0 = no, 1 = yes)	–	–	–	–	−25.031	0.089	294.816	0.565
BMI (kg/m^2^)	–	–	–	–	−1.180	0.227	−11.418	0.737
SEX * GROUP	0.039	0.151^a^	0.002	0.993^a^	3.243	0.837^a^	5.291	0.992^a^
								
**Random effects (variances)**								
INTERCEPT	0.003	–	<0.001	–	256.78	–	659,935	–
Residual variance	0.008	–	0.403	–	2600.78	–	2692.464	–
								
BIC	−236.17	–	383.79	–	1849.55	–	2819.53	–
Number of individuals	177	–	172	–	171	–	161	–
Number of couples	90	–	90	–	90	–	87	–

MOOD_d, change in state mood; RELSAT_d, change in momentary relationship satisfaction; sCORT, salivary cortisol (in ng/ml); sAA, salivary alpha-amylase (in U/min); AUCi, area under the curve with respect to increase; BMI, body mass index; Est., Estimate; BIC, Bayesian information criterion; bold effects were statistically significant on the level of p <0.05; ^a^ tested in hypothesis 2.a.

Men’s average sCORT and sAA AUCi were positive and the sCORT AUCi’s were descriptively higher than those of their female partners (**Table 2**). Trajectories were comparable between men in the DCs and NDCs group with regard to sCORT and sAA, while sAA levels were higher in DCs (**Figures 3C, D**). However, none of the tested, interaction effects were statistically significant in multilevel modeling of sCORT AUCi and sAA AUCi (both *p > *0.050, **Table 4**). Moreover, RELDUR was not a significant moderator of any SEX * GROUP effect in Models 5–8, and therefore, final models without RELDUR and its higher-order interactions were reported in **Table 4**.

### Explorative Associations of Age and Relationship Duration

Given the identified moderating role of relationship duration in women’s cortisol response, we explored its associations with other psychological and psychobiological variables in this study to gain a deeper understanding into the meaning of this finding (**Table 5**). Unsurprisingly, relationship duration was strongly related with age in all participants (*r* = −0.71). Furthermore, we found longer relationship duration to be associated with lower partnership quality (PFB) and lower actually received social support (BSSS), and correlations were stronger in depressed women (PFB: *r* = −0.41, BSSS: *r* = −0.39) than in non-depressed women (PFB: *r* = −0.21, BSSS: *r* = −0.02). Interestingly, while non-depressed women’s relationship duration was associated with a stronger increase in PAT-induced mood (*r* = 0.16) and a lower sCORT AUCi (*r* = −0.23), the opposite direction of associations was found in depressed women: Here, longer-term relationships were associated with less positive mood changes (*r* = −0.22) and a higher cortisol output (sCORT AUCi: *r* = 0.38).

**Table 5:** Explorative correlations [95% confidence intervals] for relationship duration.

	All (N = 184)	DCs—Women (N = 47)	NDCs—Women (N.=.45)
**Age**	**0.71 [0.63; 0.77]**	**0.74 [0.57; 0.84]**	**0.73 [0.55; 0.84]**
**Relationship quality (PFB)**	**−0.34 [−0.46; −0.21]**	**−0.41 [−0.21; −0.14]**	**−**0.21 [**−**0.47; 0.09]
**Social Support (BSSS)**	**−**0.26 [**−**0.39; **−**0.12]	**−0.39 [−0.61; −0.12]**	**−**0.02 [**−**0.31; 0.28]
**MOOD_d**	**−**0.08 [**−**0.22; 0.07]	**−**0.22 [**−**0.48; 0.07]	0.16 [**−**0.14; 0.43]
**sCORT AUCi**	**−**0.10 [**−**0.24; 0.04]	**0.38 [0.10; 0.60]**	**−**0.23 [**−**0.49; 0.07]

PFB, Partnership Questionnaire; BSSS, Berlin Social Support Scales—actually received social support; MOOD_d, change in state mood; sCORT, salivary cortisol (in ng/ml); AUCi, area under the curve with respect to increase; DCs, depressive couples; NDCs, non-depressive couples; bold correlations were medium or large effects (r >0.30 or r < **−**0.30).

## Discussion

### Summary and Interpretation of Findings

With the present study, we aimed at investigating the affective and psychobiological response of couples with depression in an instructed dyadic interaction setting in the lab. Couples with the female partner suffering from depression (DCs) and non-depressed controls (NDCs) were asked to perform an instructed PAT sequence that included positive and appreciative communication between romantic partners. Mood, momentary relationship satisfaction, and biological indicators of stress and arousal were repeatedly assessed during and following the task.

Our primary analyses focused on differences in PAT-induced trajectories between depressed and non-depressed women. Previous research in general populations showed that positive social interaction can increase mood and activate reward-related central nervous system mechanisms (99, 100). Social feedback from the partner, as the most relevant person to most adults, has been shown to substantially affect mood in laboratory studies and in couples’ everyday life (41, 101). We expected depressed women to benefit less from positive interaction with their partners due to social anhedonia and the usual tendency to avoid these situations (23). Increases in state mood, however, were comparable in magnitude between depressed and non-depressed women and differences were not significant. Hence, the presence of a depressive diagnosis did not lead to women evaluating the interaction as unpleasant, despite previous evidence from eye-tracking studies suggesting that depressed individuals avert positive (social) stimuli (19). In contrast, depressed women reported affective benefits from appreciative conversation with their partners. Moreover, increases in relationship satisfaction were even stronger in depressed than in non-depressed women, indicating that the engagement in positive interaction with the partner directly entailed social evaluative processes regarding the partnership. It should be noted that depressed women had the lowest baseline scores in both mood and relationship satisfaction. While this shows that the chosen outcomes were apt to clinically characterize the study groups at baseline, there is also the possibility of statistical regression-to-the-mean effects. However, these effects seem rather unlikely here, as these baseline variability was not due to extreme values or outliers but to theoretically expected differences in clinically distinguishable groups. Therefore, the findings show that depressed women’s mood and relationship satisfaction improve from participation in appreciative communication and that the PAT can reduce pre-existing baseline differences in these variables compared to non-depressed women.

As depressed women usually tend to avoid PAT-like situations, we hypothesized that the instructed (or “forced”) participation in positive communication would require high mental and affective effort and that this would transfer to a pattern of psychobiological arousal or stress response. This assumption partly received support with regard to cortisol trajectories: Depressed women showed a higher increase in cortisol in response to the PAT, but this effect was only significant if relationship duration was considered as a moderating factor. Hence, the identified increase in cortisol output was particularly pronounced for female partners in long-term relationships. sAA levels also increased over the course of the PAT in depressed women, but differences between the groups were not significant. On a descriptive level, the T1–T2 decrease in non-depressed women’s sAA may reflect adjustment to the experimental situation after initial arousal, which was not found in depressed women. Hence, the increased psychobiological arousal observed in both the sCORT and sAA trajectories in depressed women may well contain an anticipatory stress component. Taken together, these results support the idea that the unfamiliar involvement in positive couple interaction requires higher effort and leads to arousal in depressed women (particularly in longer-term relationships), but that successful engagement in the PAT offers potential affective and social benefits with regard to the partnership.

As the psychobiological arousal effects were not found independent of relationship duration, we explored associations of RELDUR with other relevant variables in order to better understand the nature of this finding. Interestingly, longer-term relationships were associated with a weaker increase in subjective mood and a stronger increase in cortisol in depressed females, while the opposite direction of associations was found in non-depressed women. Moreover, we found negative correlations between relationship duration and partnership quality (PFB) and actually received social support by the romantic partner (BSSS), particularly in depressed women. Hence, longer relationship duration was associated with impairments in marital/relationship functioning, which is consistent with previous research (14, 15). With increasing duration, couples were found to report less companionship, sexual interaction, relational satisfaction, and commitment on the one hand, and higher frequency of conflict and arguing on the other hand (102). The effect received further support by longitudinal data from a female sample showing not only a decline in relationship quality after 10 years, but also an increased risk for the later occurrence of depressive symptoms if relationship quality was initially low (16). More broadly, marital strain seems to accelerate the typical decline in general health over time (103), and HPA and SAM dysfunctions were found in partners with insecure attachment styles (104). Other studies, in contrast, reported a protective effect of relationship duration on mental health (105), but these were found only in individuals younger than 30 years. In the present study, depressed women in long-term relationships already had developed a mental disorder despite the potentially protective effect of partnership in early years of a relationship, and then showed an increased HPA activation in the PAT. As the moderating role of relationship duration was identified in exploratory analyses, inferences should be drawn cautiously and future studies should be conceptualized to directly test this effect in depressed couples.

A secondary set of analyses in this study included data from male partners. Descriptively, male partners in the DCs showed higher scores of depressiveness on average than men in the NDCs (**Figure 2A**). This is in line with previously reported findings suggesting depressive disorders to affect not only the individual, but whole social systems, particularly including romantic partnerships (5, 106). Notably, the average PHQ-9 score of *M* = 5.51 (*SD* = 4.33) for males in the DCs group would pass the cut-off for a mild depression according to common classifications (107) and *N* = 7 men had a HDRS rating ≥12. Moreover, both partners in the DCs descriptively reported lower partnership quality (PFB), less actually received social support from the partner (BSSS), and higher chronic stress (TICS) than NDCs, and sex-differences within DCs were rather neglectable (**Figures 2B–D**). Hence, DCs as an entity were not only characterized by depression-related symptoms, but also revealed further impairments in social functioning and stress when compared to NDCs. Previous research identified similar profiles in couples with depression, showing reduced quality of life, less perceived social support, higher occurrence of stressful events, and impairments in family or marital functioning (108). These comparable patterns in couple-related functioning and chronic stress may help to explain the paucity of observed sex-dependent group effects in the dyadic analyses. In fact, we did not find any significant SEX * GROUP interactions with regard to mood, relationship satisfaction or stress/arousal markers. Men in both groups improved in mood and patterns of change in RELSAT, sCORT, and SAA did not differ significantly from the female partners or from each other. While these non-significant findings may also depend on sample size and high variability in psychobiological data, they also suggest that both partners are noticeably affected by the mental disorder, and that it is worthwhile to consider the couple as an important unit in depression research and treatment. Taken together, the couple data suggest that instructed positive interaction may lead to affective and psychosocial benefits in couples with depression and encourage speculations about the usefulness of PAT-like interventions as a therapeutic tool. With the aim of challenging social anhedonia behavior and reduced attempts to approach socially rewarding situations in depression (5, 18, 22, 23, 27), couples might be instructed to use positive feedback under a therapist’s supervision.

### Limitations

A major strength of this research was the integration of complex data within a comprehensive bio-psycho-social approach to the study of positive interaction in depressed couples. However, the study faced a number of limitations which need to be considered. First, DCs on average were 5.8 years older than NDCs. We became aware of this imbalance between groups at an early stage of the study and identified the high percentage of participants in a students’ age in the NDCs as a possible reason. While the financial incentive may have been appealing particularly for younger, healthy subjects, DCs’ participation in the SIDE studies may have been driven more by the opportunity to benefit from the subsequent CBCT^®^ couple therapy (73). Despite the development of strategies to recruit older couples in the NDCs group (e.g. by offering incentives such as mindfulness courses free of charge and by tailoring the advertising strategy to older participants), we were unable to eliminate this possible source of bias completely. As we intended our findings to remain as unbiased as possible, all subsequent analyses were statistically adjusted for age. Second, to test whether the PAT (instead of conflict conversations or the TSST) would result in a psychobiological stress response in depressed individuals was a novel, previously untested paradigm. It is reasonable to assume that even in depression, stressfulness of positive conversation is lower than a “classical” stress task and that increases in stress biomarkers may rather represent global arousal. In addition, the identification of relationship duration as a potential moderator in the cortisol response was data-driven and the reported findings should therefore be considered exploratory. More confirmatory research is needed to verify these results. Moreover, residuals of the model fitted to predict RELSAT_d were found to be leptokurtic compared to a normal distribution and only moderate overall model fits were observed for models predicting both MOOD_d and RELSAT_d. We decided to accept these limitations given the fact that no significant effects were observed, and the danger of reporting false positive results could thus be neglected. Lastly, inferences on the potential therapeutic benefits of the PAT need to be drawn cautiously, as we did not implement a randomized control group for a direct evaluation of effectiveness (i.e. depressed couples who were assessed but did not participate in the PAT).

### Conclusions

Contrasting expectations based on attentional bias and social anhedonia reported in depression, we found depressed women to respond to and benefit from a positive and appreciative interaction with their romantic partners with regard to state mood and momentary relationship satisfaction. At the same time, depressed women had a higher cortisol output in the PAT than healthy controls, particularly if they were in a longer-term relationship. Relationship duration in depressed women was associated with lower relationship quality, less social support, weaker PAT-induced mood increases and stronger increases in cortisol. Male partners of depressed women reported increased distress with regard to depressiveness, social support and chronic stress, and PAT-related trajectories did not significantly differ between men and women, favoring the considerations of the couple as an important unit in depression research and treatment.

Instructed engagement in positive couple interaction, which depressed women usually tend to avoid, may have required high internal resources and led to increased psychobiological arousal, before offering the chance to emotionally and socially benefit in case of successful completion. While these findings encourage speculations about the therapeutic application of instructed partnership appreciation, more research is needed to evaluate the effectiveness of such interventions, for instance in randomized trials using ecological momentary assessments or to clarify the moderating role of relationship duration.

## Data Availability Statement

The datasets presented in this study can be found in online repositories. The names of the repository/repositories and accession number(s) can be found below: heiDATA repository: https://doi.org/10.11588/data/UNWRFN.

## Ethics Statement

The studies involving human participants were reviewed and approved by Ethics Committee of the Medical Faculty at Heidelberg University. The patients/participants provided their written informed consent to participate in this study.

## Author Contributions

CA-R, MJ, and BD designed the research study. CA-R, MW, and FW performed the study. MS and MW analyzed the data. MW, MS, and BD drafted the first version of the manuscript. All authors contributed to the article and approved the submitted version. All authors agree to be accountable for the content of the work.

## Funding

MW and MJ received funding by the Physician-Scientist-Program of the Medical Faculty at Heidelberg University. The study was further supported by “Stiftungen und Preise” of the Medical Faculty at Heidelberg University as well as by the Mind and Life Organization through two Francisco J. Varela Awards granted to CA-R. CA-R was further supported by the Olympia Morata Program of Heidelberg University.

## Conflict of Interest

The authors declare that the research was conducted in the absence of any commercial or financial relationships that could be construed as a potential conflict of interest.

The handling editor declared a shared affiliation with the authors, MW, MS, FW, CA-R and BD, at time of review.
