# Supplementary material for: Instructed Partnership Appreciation in Depression: Effects on Mood, Momentary Relationship Satisfaction, and Psychobiological Arousal
Source: Front Psychiatry. 2020 Jul 30;11:701. doi: 10.3389/fpsyt.2020.00701 (PMC7409945; doi:10.3389/fpsyt.2020.00701)
Supplement: Supplementary file 1 [file DataSheet_1.pdf]

***PAT INSTRUCTIONS***

** READ OUT THE FOLLOWING WORDING AS LITERATE AS POSSIBLE:*

In the following, I would like to ask you to talk about your partnership for about 10 minutes.
For this purpose, I will read out the following instructions:

** INSTRUCTION:*
Right in front of you, you will find a list of attributes/themes that can arouse positive feelings in a partnership. Please look through the **items that are relevant to you,** and also look for additional attributes which you perceive as positive in your partnership, or where you have made positive experiences recently.
Please **be appreciative of your partner with regard to the chosen attributes/themes** and focus on specific behavior he/she has been showing in the past two months. If nothing more occurs to you regarding the chosen attributes/themes, try to remember beautiful moments in your partnership and tell your partner what affected you most about it.

** GIVE TIME FOR WATCHING THE LIST*

** PRESENT THE FOLLOWING GENERAL INFORMATION AFTERWARDS:*
- You are free to choose the order of the listed items
- Please note that you do not have to talk about all listed items. Concentrate on the **most relevant attributes/themes**
- If you realize at any point that you are talking about something negative, please try to **switch back to a positive attribute/theme**

Do you have any further questions?
Could you briefly repeat in your own words your understanding of the following task?

** MAKE SURE THE COUPLE UNDERSTOOD THAT THEY ARE HAVING A* ***MUTUAL CONVERSATION*** *AND THAT NOT ONE PARTNER IS SUPPOSED TO SPEAK AND THE OTHER TO LISTEN*

I will now leave the room and return again after 10 minutes.

**LIST OF POSITIVE ATTRIBUTES/THEMES**

In the following list, you can find attributes/themes that can arouse positive feelings in a partnership. Please look through the **items that are relevant to you,** and also look for additional attributes which you perceive as positive in your partnership, or where you have made positive experiences recently.

Please **be appreciative of your partner with regard to the chosen attributes/themes** and focus on specific behavior he/she has been showing in the past two months. If nothing more occurs to you regarding the chosen attributes/themes, try to remember beautiful moments in your partnership and tell your partner what affected you most about it.

| | |
|---|---|
| 1. | Partner’s attention |
| 2. | Satisfaction with career |
| 3. | Sharing of household work |
| 4. | Shared beliefs about parenting |
| 5. | Shared leisure time |
| 6. | Friends |
| 7. | Partner’s patience |
| 8. | Peace of mind with regard to financial situation |
| 9. | Attractiveness |
| 10. | Trust |
| 11. | Attachment |
| 12. | Giving personal freedom |
| 13. | Sexuality |
| 14. | Loyalty |
| 15. | Family/relatives |
| 16. | Tolerance of partner’s personal habits |
| 17. | Communication / collective discussions |
| 18. | Desire to have children / family planning |
| 19. | Supporting the partner |
| 20. | Partner’s generosity |
| 21. | Health / plans for the future |
| 22. | Mutual support in times of crisis |
| 23. | Tenderness |
| Others: | |
